# Ultrasound‐Guided Modified Thoracoabdominal Nerve Block Through Perichondrial Approach for Postoperative Analgesia Management in Living Liver Donors: A Randomized, Prospective, Controlled Study

**DOI:** 10.1111/ctr.70224

**Published:** 2025-09-02

**Authors:** Hande Gungor, Ayşe Ince, Bahadir Ciftci, Birzat Emre Gölboyu, Mert Asici, Pelin Karaaslan, Tumay Uludag Yanaral

**Affiliations:** ^1^ Department of Anesthesiology and Reanimation Istanbul Medipol University Istanbul Turkey; ^2^ Department of Anatomy Istanbul Medipol University Istanbul Turkey; ^3^ Department of Anesthesiology and Reanimation Katip Çelebi University Izmir Turkey

**Keywords:** liver transplantation, living donor hepatectomy, M‐TAPA block, pain management, regional anesthesia, ultrasonography

## Abstract

**Background:**

Optimal postoperative pain management in living donor hepatectomy remains challenging, with conventional methods showing limitations. This study evaluated the efficacy and safety of ultrasound‐guided modified thoracoabdominal nerve block through a perichondrial approach (M‐TAPA) compared to conventional pain management in living donor hepatectomy patients.

**Methods:**

In this prospective, randomized, controlled, single‐blind study conducted between April 2024 and January 2025, 50 ASA I‐II patients undergoing living donor right hepatectomy were randomly allocated to either the M‐TAPA group (*n* = 25, receiving ultrasound‐guided M‐TAPA block plus standard analgesia) or the Control group (*n* = 25, receiving conventional pain management only). The primary outcome was postoperative opioid consumption during the first 48 h. Secondary outcomes included pain scores, rescue analgesia requirements, and complications.

**Results:**

The M‐TAPA group showed significantly lower median total fentanyl consumption (*p* = 0.002) and reduced need for rescue analgesia (*p* = 0.011) compared to the Control group. Both static and dynamic Numeric Rating Scale pain scores were significantly lower in the M‐TAPA group across all time points (*p* < 0.001). Although the M‐TAPA group showed a trend toward reduced nausea incidence (*p* = 0.066), other side effects were comparable between groups. No M‐TAPA block–related complications were reported.

**Conclusions:**

Ultrasound‐guided M‐TAPA block provides effective postoperative pain management in living donor hepatectomy, demonstrating significant reductions in opioid consumption and pain scores without increasing complications. These findings suggest MTAPA could be a valuable component of enhanced recovery protocols in living donor liver transplantation programs.

**Trial Registration:**

ClinicalTrials.gov identifier: NCT06300372

## 1 Introduction

Optimal management of postoperative pain in patients undergoing living donor hepatectomy is crucial for enhancing recovery, promoting early mobilization, and ensuring donor satisfaction. Although opioid‐based strategies have been fundamental in pain management for these complex procedures, they are associated with numerous complications (including sedation, respiratory depression, nausea, vomiting, and urinary retention) that can significantly impede recovery [1]. Although central neuraxial blocks have long been considered the gold standard for major abdominal surgeries, they face limitations in the context of liver resection due to potential coagulopathy and difficulty of application and complications (cord injury, hypotension, lower extremity weakness, urinary retention, etc.) [2].

In light of these challenges, there is a growing interest in interfascial plane blocks that can provide effective pain control while minimizing systemic complications [3, 4, 5, 6]. The modified thoracoabdominal nerves block through the perichondrial approach (M‐TAPA) has emerged as a promising alternative analgesic technique in abdominal surgeries. M‐TAPA targets both the anterior and lateral branches of the thoracoabdominal nerves (T6‐T12) by injecting local anesthetic into the lower surface of the perichondrium, offering a more extensive and tailored approach compared to the classic TAPA block [7, 8, 9].

Previous clinical studies suggest that M‐TAPA may provide better coverage of the upper abdominal wall and midline incisions of the abdomen, making it particularly suitable for procedures such as open liver resections, including donor hepatectomy [10, 11, 12, 13]. However, its efficacy in this specific context remains to be fully evaluated.

This study aimed to assess the efficacy and safety of ultrasound‐guided M‐TAPA block in patients undergoing donor hepatectomy, comparing it to conventional pain management strategies in terms of postoperative pain control and overall analgesia management. Our hypothesis was that M‐TAPA block would provide enhanced analgesia compared to conventional analgesia strategies.

## 2 Materials and Methods

### 2.1 Study Design

**FIGURE 1:**
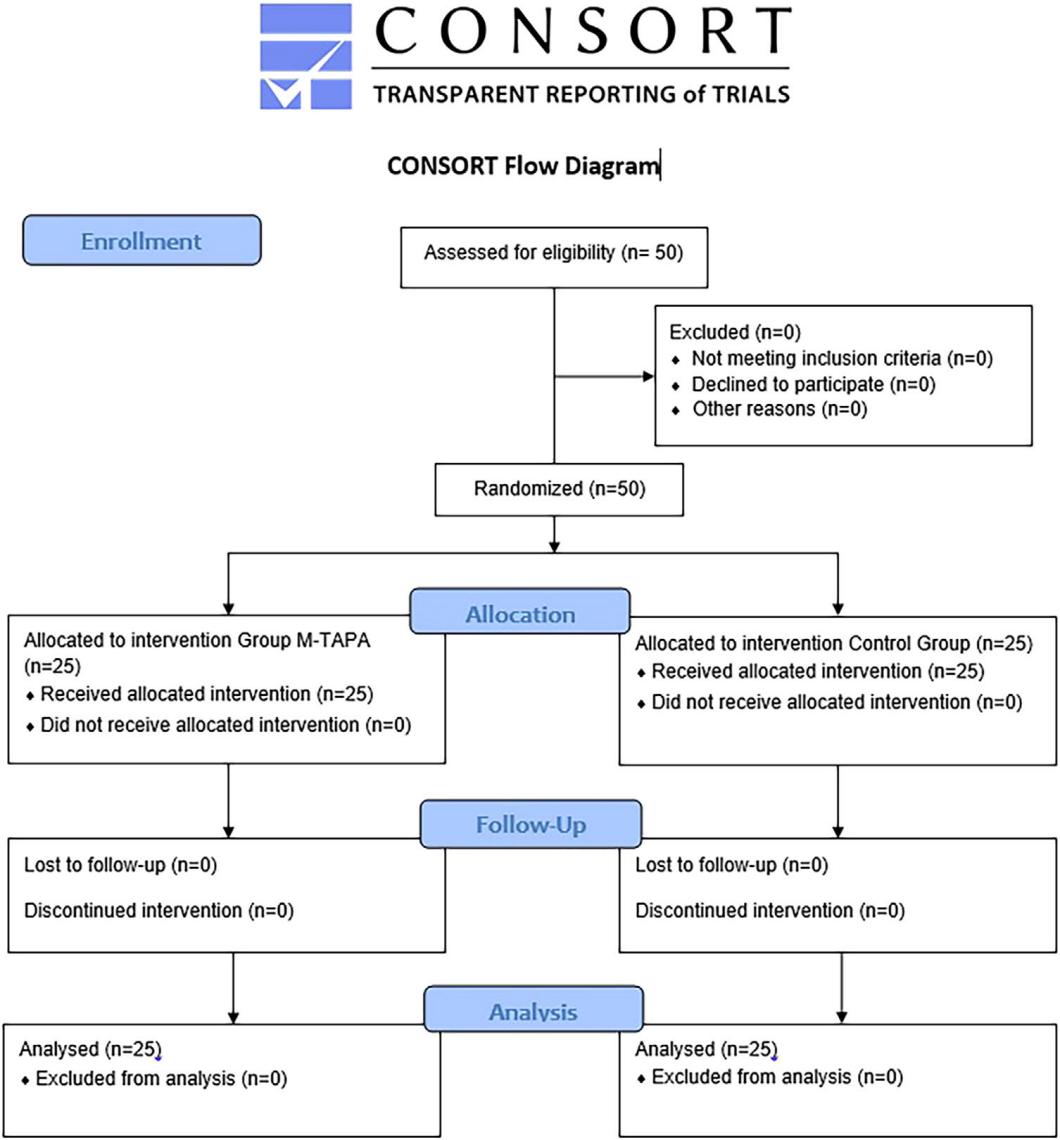
CONSORT flow diagram of the study.

This was a prospective, randomized, controlled, single‐blind comparative study. The study was conducted at Istanbul Medipol University Mega Hospital Complex, a tertiary care center, between April 2024 and January 2025. The study was approved by the Institutional Ethics and Research Committee of Istanbul Medipol University, Istanbul, Turkey (Approval No: 675) on October 8, 2023 and then registered at a clinical trial on March 3, 2024. Following verbal explanation, written informed consent was obtained from each participant as a record of their agreement to partake in the study. All procedures were conducted in accordance with applicable guidelines and regulations. The study adhered to the Consolidated Standards of Reporting Trials (CONSORT) guidelines for reporting (Figure 1).

Eligible participants were American Society of Anesthesiologists (ASA) classes I‐II patients aged 18–65 who were scheduled for elective donor right hepatectomy under general anesthesia. Exclusion criteria included coagulopathy, allergy to local anesthetics or opioid agents, infection at the block area, refusal of the procedure, or inability to use the patient‐controlled analgesia (PCA) device.

The donor population included direct relatives (siblings or offspring), extended family members (e.g., cousins), and nonrelated altruistic donors. Hepatic resections were tailored for each donor–recipient pair, considering anatomical suitability, donor safety, and recipient requirements. All donors in this study underwent right hepatectomy.

### 2.2 Grouping and Randomization

Eligible patients were randomly allocated to two groups before they arrived at the operating room: M‐TAPA group (*n* = 25), receiving ultrasound‐guided M‐TAPA block plus standard analgesia; and Control group (*n* = 25), receiving conventional pain management.

The randomization was concealed using sealed opaque envelopes. We chose not to use sham blocks due to ethical concerns regarding unnecessary invasive procedures in the control group. To maintain scientific rigor, we ensured that the assessment team was completely blinded to group assignments when collecting all outcome data. All patients had adhesive dressings applied to the potential block sites, regardless of group allocation. Postoperative outcomes were evaluated and recorded by a blinded pain nurse anesthetist who was unaware of group allocation.

### 2.3 General Anesthesia and Surgical Procedure

In the operating room, all patients were monitored with electrocardiography, noninvasive blood pressure monitoring, and pulse oximetry. Standardized anesthesia induction was performed with propofol 2–2.5 mg/kg, fentanyl 1–1.5 µg/kg, and rocuronium 0.6 mg/kg intravenously (iv).

Anesthesia maintenance was achieved with sevoflurane (1–1.2 MAC) in a mixture of oxygen and fresh air and continuous infusion of vecuronium (0.8–1 µg/kg/min) and remifentanil (0.1–0.3 µg/kg/min). Additionally, 8 mg of ondansetron was administered iv to prevent nausea and vomiting.

All patients underwent living donor hepatectomy through the same open surgical procedure (midline incision) by the same surgical team. Postoperatively, patients with adequate spontaneous breathing were extubated and transferred to the intensive care unit.

### 2.4 Perioperative Analgesia Regimen

All patients in both groups received conventional multimodal analgesia as per our institutional protocol. For preemptive analgesia, morphine 0.05 mg/kg and ibuprofen 400 mg iv were administered to all patients before the surgical incision. Ibuprofen 400 mg, tramadol 1 mg/kg, and meperidine 0.5 mg/kg were administered 30 min before the end of surgery.

### 2.5 M‐TAPA Procedure

**FIGURE 2:**
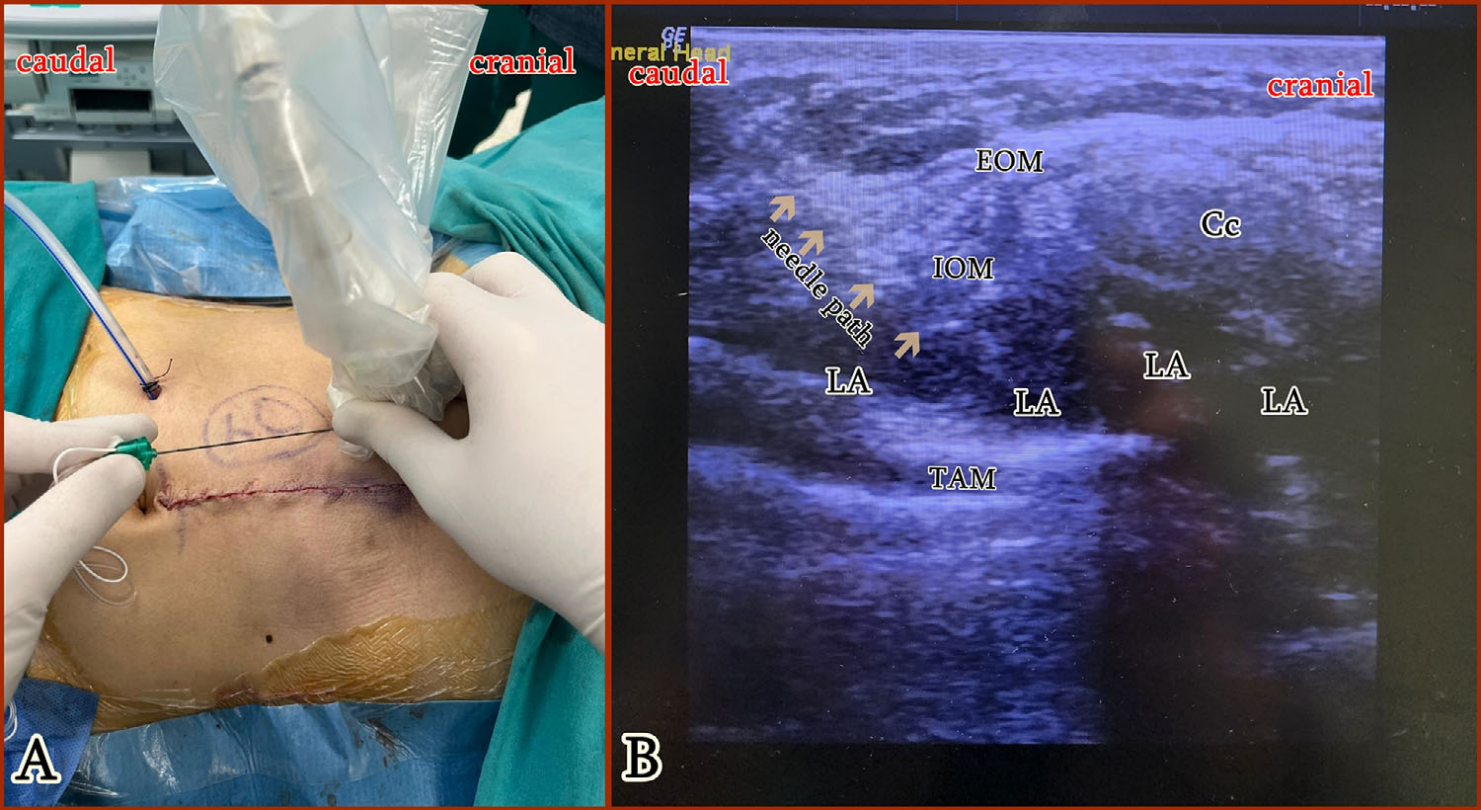
A. Patient and probe position during M‐TAPA block. B. Sonographic visualization of M‐TAPA block. 10th costal cartilage and spread of local anesthetic are seen. The arrows indicate the needle path. CC: costal cartilage, EOM: external oblique muscle, IOM: internal oblique muscle, LA: local anesthetic, P: perichondrium, TAM: transversus abdominis muscle.

In addition to the standard analgesia, patients in the Block group received bilateral ultrasound‐guided M‐TAPA blocks. The procedure was performed at the end of the surgery, before extubation, using a high‐frequency linear ultrasound. Because of the long duration of hepatectomy surgery, we performed the M‐TAPA block at the end of surgery to maximize block efficacy during the postoperative period. With the patient in the supine position, the probe was placed longitudinally over the costal margin, at the level of the 9th to 10th ribs, for visualization of the abdominal muscles, and identification of the perichondrium (Figure 2A). Using an in‐plane technique, the block needle (22 G x 80 mm, Braun Stimuplex Ultra 360, Germany) was advanced in the caudo–cranial direction. After confirming the correct location by injecting 5 mL of saline into the lower surface of the perichondrium, 30 mL of 0.25% bupivacaine between the internal oblique muscle and transversus abdominis muscle was administered (Figure 2B). The same procedure was performed on the other side. A total of 60 mL of 0.25% bupivacaine (30 mL on each side) was administered. No regional block or local infiltration techniques were applied to the control group.

### 2.6 Postoperative Multimodal Analgesia Protocol and Evaluation of Pain

In the postoperative period, all patients in both groups received acetaminophen (1000 mg) iv every 8 h. All patients were attached to an iv fentanyl PCA device according to the protocol: 10 µg/mL fentanyl, a 0.35 µg/kg bolus, a 15‐min lockout time without a basal dose, and a maximum hourly limit of 100 µg.

Blinded investigators utilized the Numeric Rating Scale (NRS) (0 = no pain; 10 = the most severe pain felt) to assess postoperative pain intensity levels at rest (static) and during movement (dynamic) across multiple time points: 0, 2nd, 4th, 8th, 12th, 24th, and 48th hours in the postoperative period. If the NRS score was ≥ 4/10, 0.5 mg/kg iv meperidine was used as rescue analgesia.

### 2.7 Outcomes

The primary outcome of this study was to compare the postoperative opioid (fentanyl) consumption from the PCA device between the M‐TAPA and control groups during the first 48 h after surgery. Secondary outcomes included assessment of postoperative pain scores, need for rescue analgesia, and incidence of opioid‐related side effects.

### 2.8 Power Analyses

The sample size of the study was calculated using the G*Power program (V.3.1.9). The primary aim in this study was to compare postoperative opioid (fentanyl) consumption from the PCA device. In the preliminary study, which included eight patients each, it was observed that the postoperative opioid consumption was 51 µg in the M‐TAPA group and 110 µg in the control group. Standard deviations were determined as 39 and 62. Considering *α* error 0.05 and *β* error 0.05, the number of patients to be included in each group was determined to be 22 with 95% power. Considering possible dropouts, we decided to include at least 25 patients per group.

### 2.9 Statistical Analyses

The shapes of the distributions of the variables in this study were assessed by using the Shapiro–Wilk test, whether the observation is normal or skewed. In cases in which the test results have indicated that the data were normally distributed, the data were detailed with mean ± SD and analyzed by an independent samples *t*‐test to compare groupwise differences in the outcome parameters. For the continuous data, which yielded the nonparametric dispersion, the median and IQR were detailed and analyzed with the Mann–Whitney *U* test to observe the groupwise differences. In our study, the statistical significance threshold was *p* value < 0.05. Statistical analyses were conducted using SPSS V.25 (SPSS, Chicago, Illinois, USA).

## 3 Results

The study encompassed 50 donors undergoing living donor hepatectomy, equally divided between the M‐TAPA group (*n* = 25) and Control group (*n* = 25). All assessed donors met the inclusion criteria and were subsequently enrolled. Table 1 presents the demographic data and surgical details, revealing no significant disparities between the groups in terms of demographics or durations of surgery and anesthesia.

**TABLE 1:** Comparison of demographic data and duration times of surgery and anesthesia.

	M‐TAPA group (*n* = 25)	Control group (*n* = 25)	*p*
**Age**	35 (24–40)	33 (26–41)	^*^ *0.961*
**Gender (M/F)**	16/9	13/12	^†^ *0.254*
**ASA (I/II)**	15/10	11/14	^†^ *0.258*
**Height (cm)**	172 (159–180)	170 (160–175)	^*^ *0.876*
**Weight (kg)**	73 (66–82)	75 (67–89)	^*^ *0.313*
**Duration of surgery (min)**	245 (220–280)	265 (240–290)	^*^ *0.111*
**Duration of anesthesia (min)**	305 (273–340)	325 (300–355)	^*^ ***0.057***

Values are expressed as median (percentiles 25–75) or number.

*p* values were italicized and values that are written in bold represent statistical significance.

Abbreviations: ASA, American Society of Anesthesiologist; cm, centimeter; F, female; kg, kilogram; M, male; min, minutes.

* *p* value is obtained with Mann–Whitney *U* test.

^†^ *p* value is obtained with Pearson's *χ* ^2^ test (*n*).

Analysis of postoperative pain management revealed notable differences between the groups. The M‐TAPA group demonstrated significantly reduced fentanyl consumption across most time intervals, with a markedly lower median total fentanyl usage (60 vs. 120 µg in the Control group, *p* = 0.002). Furthermore, the M‐TAPA group exhibited a substantially decreased need for rescue analgesia. Specifically, meperidine consumption as rescue medication was significantly lower in the M‐TAPA group (0 mg vs. 30 mg in the Control group, *p* = 0.048). The proportion of patients requiring rescue analgesia was also significantly smaller in the M‐TAPA group (8 vs. 17 patients, *p* = 0.011). These findings are detailed in Table 2.

**TABLE 2:** The comparison of postoperative opioid consumptions (fentanyl) and the use of rescue analgesia (meperidine) between groups.

	M‐TAPA group (*n* = 25)	Control group (*n* = 25)	*p*
**PCA 0–8 h (µg)**	20 (0–30)	50 (25–75)	***0.001***
**PCA 8–16 h (µg)**	20 (0–25)	20 (0–25)	*0.367*
**PCA 16‐–24 h (µg)**	0 (0–25)	20 (0–30)	***0.122***
**PCA 24–48 h (µg)**	0 (0–0)	20 (0–50)	***0.001***
**PCA total (µg)**	60 (20–85)	120 (50–150)	***0.002***
**Rescue analgesia (Y/N)**	8/17	17/8	^†^ ***0.011***
**Rescue dose (mg)**	0 (0–40)	30 (0–40)	***0.048***

Data are expressed as median.

*p* value is obtained with Mann–Whitney *U* test median (percentiles 25–75).

*p* values were italicized and values that are written in bold represent statistical significance.

Abbreviations: µg, microgram; min, minutes; N, no; PCA, patient‐controlled analgesia; Y, yes (indicates the number of the patients that used rescue analgesia); N/A, Not Applicable.

^†^ *p* value is obtained with Pearson's *χ* ^2^ test (*n*).

Pain assessment using the NRS yielded consistent results favoring the M‐TAPA group. Both static and dynamic NRS scores were significantly lower in the M‐TAPA group compared to the control group across all time points throughout the 48‐h postoperative period (*p* < 0.001 for all comparisons). This indicates enhanced pain control in the M‐TAPA group, both at rest and during movement, as shown in Table 3.

**TABLE 3:** Comparisons of static and dynamic NRS assessment between groups.

	M‐TAPA group (*n* = 25)	Control group (*n* = 25)	*p*
**At rest**			
1st hour	0 (0–2)	2 (2–3)	***0.001***
2nd hour	0 (0–2)	2 (2–4)	***0.001***
4th hour	0 (0–1)	2 (2–3)	***0.001***
8th hour	0 (0–1)	3 (2–3)	***0.001***
16th hour	0 (0–0)	2 (2–3)	***0.001***
24th hour	0 (0–0)	2 (2–3)	***0.001***
48th hour	0 (0‐0)	2 (1–2)	***0.001***
**On movement**			
1st hour	0 (0–4)	3 (2–4)	***0.003***
2nd hour	2 (0–3)	3 (3–5)	***0.001***
4th hour	0 (0–2)	3 (2–4)	***0.001***
8th hour	0 (0–2)	3 (2–3)	***0.001***
16th hour	0 (0–1)	3 (2–3)	***0.001***
24th hour	0 (0–0)	2 (2–3)	***0.001***
48th hour	0 (0–0)	2 (1–2)	***0.001***

Data are expressed as median (percentiles 25–75).

*p* values were italicized and values that are written in bold represent statistical significance.

Abbreviation: NRS, Numeric Rating Pain Scale.

Regarding postoperative side effects, a trend toward reduced incidence of nausea was observed in the M‐TAPA group, though this difference did not achieve statistical significance (2 vs. 7 patients, *p* = 0.066). Incidences of vomiting and itching were comparable between the groups, with no significant differences noted. These observations are summarized in Table 4. Importantly, no complications associated with the M‐TAPA block were reported throughout the study period.

**TABLE 4:** Comparison of the incidence of side effects between groups.

	M‐TAPA group (*n* = 25)	Control group (*n* = 25)	*p*
**Nausea (Y/N)**	2/23	7/18	*0.066*
**Vomiting (Y/N)**	2/23	4/21	*0.384*
**Itching (Y/N)**	1/24	4/21	*0.157*

*p* value is obtained with Pearson's *χ* ^2^ test (*n*).

Abbreviations: N, No; Y, yes.

These results suggest that the M‐TAPA block offers enhanced postoperative pain management in living donor hepatectomy, characterized by reduced opioid consumption and decreased need for rescue analgesia, improved pain scores, and a trend toward fewer side effects.

## 4 Discussion

According to our results, the M‐TAPA block provided significantly better postoperative pain management compared to conventional pain management strategies in living donor hepatectomy patients. In terms of opioid consumption, the M‐TAPA group showed significantly lower fentanyl requirements (60 µg vs. 120 µg, *p* = 0.002) throughout the postoperative period. Additionally, rescue analgesic consumption was markedly reduced in the M‐TAPA group, with significantly lower meperidine requirements (0 mg vs. 30 mg, *p* = 0.048) and fewer patients needing rescue analgesia overall (8 vs. 17 patients, *p* = 0.011). These findings suggest that the enhanced postoperative pain control achieved with the M‐TAPA block can be attributed to its ability to effectively target the key sources of pain following living donor hepatectomy.

Pain following living donor hepatectomy has a complex, multifactorial origin, with multiple contributors to postoperative pain intensity. The primary sources include somatic pain from the extensive right subcostal incision and midline incision (approximately 15–20 cm), deep visceral pain from liver parenchymal resection and manipulation, inflammatory pain from surgical trauma and tissue handling, and musculoskeletal pain from surgical retraction and disruption of the abdominal wall integrity.

This clinical challenge is well‐documented in controlled studies. Butt et al. conducted a multicenter prospective study of 271 donors finding that 21% experienced clinically significant pain during follow‐up [14]. Mandell et al. reported that Living donor liver transplantation (LDLT) donors had significantly higher pain scores compared to other surgical populations, requiring more intensive analgesia strategies [15]. Regarding negative outcomes from opioid use in these patients, Erdogan et al. documented LDLT donors requiring high opioid doses (mean 1276.8 µg remifentanil and 73 mg morphine in control group) during the first 48 postoperative hours, resulting in significant complications, including ileus, nausea/vomiting, and respiratory depression [6]. Moreover, studies have shown that inadequately managed acute postoperative pain may progress to chronic pain syndrome. De Carlis et al. reported that in donors who experienced recipient death after transplant, persistent negative outcomes in terms of fatigue and chronic pain were documented [16]. Dew et al. found that among 178 donors with pain or fatigue issues, 31% experienced both symptoms simultaneously during their 2‐year follow‐up period [17]. These findings collectively underscore the critical importance of effective perioperative pain management to prevent both immediate discomfort and potential long‐term complications.

Traditional pain management strategies that heavily rely on opioids present significant challenges due to various complications that can impede recovery and compromise enhanced recovery protocols, including respiratory depression, postoperative nausea and vomiting, delayed bowel function recovery, and prolonged hospital stays. Recent advancements in perioperative care have addressed these challenges by emphasizing multimodal analgesic approaches, which integrate various pain management strategies combining regional anesthetic techniques with systemic analgesics [18]. In line with these principles, our study demonstrated a reduction in opioid consumption in the M‐TAPA group compared to the control group. Building upon these observations, our analysis revealed significantly lower pain scores in the M‐TAPA group compared to the control group, with a clinically meaningful reduction on the NRS at all postoperative time points. Previous studies have demonstrated that fascial plane blocks provide optimal pain management after hepatectomy surgery [19, 20, 21], and M‐TAPA may represent a new and effective alternative regional anesthesia technique in this patient group due to its ease of performance and target dermatome area.

To address these significant pain management challenges in LDLT, several regional anesthesia approaches have been investigated. Koul et al. [2] investigated thoracic epidural analgesia in 104 donors, finding it reduced opioid requirements by approximately 40% [22]. Erdogan et al. demonstrated that transversus abdominis plane blocks reduced morphine consumption by 42% and shortened hospital stay [6]. Building on these established approaches, our investigation into M‐TAPA represents an evolution in the quest for optimal regional anesthesia techniques for this challenging patient population.

The technical advantages of M‐TAPA stem from its unique anatomical approach and injection technique. The M‐TAPA block, first described by Tulgar et al. in 2019, represents an advancement over traditional regional anesthesia techniques for abdominal surgeries [22]. Unlike conventional TAP blocks, M‐TAPA specifically targets the lower surface of the costal cartilage, providing wider dermatomal coverage (T6‐T12) and affecting both anterior and lateral nerve branches of thoracoabdominal nerves. This dual coverage results in more extensive analgesic spread compared to rectus sheath blocks, which primarily affect only anterior branches [23]. The efficacy of M‐TAPA is enhanced by the anatomical characteristics of the abdominal wall, where relatively less vascularization leads to slower local anesthetic clearance and prolonged analgesic effects. Tanaka et al. reported high success rates with the M‐TAPA block, demonstrating the cranial spread of local anesthetic through the anatomical feature they termed the “tunnel structure” [12]. Based on these anatomical properties, while our study used an upper midline incision, M‐TAPA may be effective for right subcostal approaches due to its T6‐12 dermatomal distribution, which corresponds well to subcostal innervation. The block's ability to reach both anterior and lateral nerve branches through a single injection location suggests potential suitability for various hepatectomy incision types.

When comparing M‐TAPA to alternative blocks for pain management following hepatectomy, several distinctions emerge. The external oblique intercostal (EOI) block and subcostal TAP block are valid alternatives for pain management following hepatectomy. The EOI block targets the lateral cutaneous branches while covering similar dermatomes [24]. The subcostal TAP block provides similar coverage [25]; however, M‐TAPA offers advantages in terms of ultrasound visualization and ease of application. The ability of M‐TAPA to reach T6‐T12 dermatomes from a single injection site allows for more comprehensive analgesia, especially beneficial for the extensive surgical field in hepatectomy.

Another practical advantage of M‐TAPA relates to patient positioning requirements. Although erector spinae plane block (ESPB) provides effective analgesia after living liver donor surgery [3, 4, 5, 26], it requires lateral positioning during placement. M‐TAPA is performed in supine position without any positioning restrictions. When compared to ESPB, M‐TAPA provides more comprehensive dermatomal coverage and affects both anterior and lateral cutaneous branches, whereas ESPB primarily targets posterior branches. Additionally, M‐TAPA offers more consistent anterior abdominal wall coverage compared to ESPB blocks, making it particularly valuable for abdominal surgical procedures in which comprehensive analgesia is required.

The safety profile of the M‐TAPA block was remarkably favorable, with no block‐related complications reported. This finding aligns with the original M‐TAPA technique description by Tulgar et al. for various abdominal surgeries and reflects the ongoing evolution of regional anesthesia techniques in liver transplantation [24].

Finally, here some questions come into mind. What the level of expertise needed to perform an M‐TAPA block? Can any anesthesiologist do an M‐TAPA block or is this recommended for someone who is fellowship‐trained or proven to be highly proficient at nerve blocks? Regarding the learning curve, the M‐TAPA block can be performed by anesthesiologists with basic ultrasound skills and experience in fascial plane blocks, though fellowship training is not mandatory. The relative ease of the block stems from its superficial anatomical location, the easily identifiable costal cartilage structure on ultrasound imaging, and the limited structures at risk. However, successful implementation requires the operator to correctly identify the costal margin and abdominal muscles, precisely guide the needle in‐plane to the fascial plane, and evaluate appropriate spread of the local anesthetic. Safety concerns include risks of peritoneal penetration, intravascular injection, and inadequate block coverage. For beginners, mentored practice followed by gradual progression to independent application is the most appropriate approach.

Our study had some limitations that warrant acknowledgment. First, we used a fixed volume of local anesthetic (30 mL) for M‐TAPA. Different findings may be obtained with different volumes, and future dose‐finding studies would be valuable. Second, we performed single‐shot M‐TAPA rather than using a continuous infusion via a catheter. It is possible that pain scores or opioid consumption levels could have been different with continuous infusion techniques. Third, our sample size was relatively small, and thus studies with larger sample sizes are needed to confirm our findings. Lastly, we did not evaluate the dermatomal blockade in patients after the M‐TAPA block, which would have provided additional insight into the duration of effect.

## 5 Conclusions

In conclusion, according to our results, M‐TAPA is a promising technique for postoperative pain management in living donor hepatectomy. It appears to offer effective analgesia with a favorable side effect profile and potential opioid‐sparing benefits. These findings could have significant implications for enhancing recovery protocols in living donor liver transplantation programs. However, further research is needed to fully establish the role of M‐TAPA in this context and to optimize its application.

## Author Contributions

All of the authors made substantial contributions to conception and design, or acquisition of data, or analysis and interpretation of data; they have been involved in drafting the manuscript or revising it critically for important intellectual content; have given final approval of the version to be published.

## Ethics Approval and Consent to Participate

This study has been approved by Istanbul Medipol University Ethics and Research Committee (Approval No: 675, dated 10.08.2023). Written informed consent was obtained from the participants. All methods were carried out in accordance with relevant guidelines and regulations.

## Conflicts of Interest

The authors declare no conflicts of interest.

## Data Availability

The datasets generated and/or analyzed during the current study are not publicly available, but are available from the corresponding author on reasonable request. The data that support the findings of this study are available from the corresponding author upon reasonable request.
